# Live Imaging of Influenza Infection of the Trachea Reveals Dynamic Regulation of CD8+ T Cell Motility by Antigen

**DOI:** 10.1371/journal.ppat.1005881

**Published:** 2016-09-19

**Authors:** Kris Lambert Emo, Young-min Hyun, Emma Reilly, Christopher Barilla, Scott Gerber, Deborah Fowell, Minsoo Kim, David J. Topham

**Affiliations:** 1 David H. Smith Center for Vaccine Biology & Immunology, University of Rochester School of Medicine and Dentistry, Rochester, New York, United States of America; 2 Department of Microbiology & Immunology, University of Rochester School of Medicine and Dentistry, Rochester, New York, United States of America; 3 Department of Surgery, University of Rochester School of Medicine and Dentistry, Rochester, New York, United States of America; Erasmus Medical Center, NETHERLANDS

## Abstract

During a primary influenza infection, cytotoxic CD8+ T cells need to infiltrate the infected airways and engage virus-infected epithelial cells. The factors that regulate T cell motility in the infected airway tissue are not well known. To more precisely study T cell infiltration of the airways, we developed an experimental model system using the trachea as a site where live imaging can be performed. CD8+ T cell motility was dynamic with marked changes in motility on different days of the infection. In particular, significant changes in average cell velocity and confinement were evident on days 8–10 during which the T cells abruptly but transiently increase velocity on day 9. Experiments to distinguish whether infection itself or antigen affect motility revealed that it is antigen, not active infection per se that likely affects these changes as blockade of peptide/MHC resulted in increased velocity. These observations demonstrate that influenza tracheitis provides a robust experimental foundation to study molecular regulation of T cell motility during acute virus infection.

## Author Summary

Influenza virus infects the cells that line the trachea and lung airways. Virus-specific cytotoxic (cell killing) T cells are the first line of adaptive immunity responsible for elimination of infected cells. We studied the cell movement, or motility, of these T cells responding to infection in the mouse trachea. Multiphoton live imaging was used to observe the cells in real time in intact tissue and measure their movement both quantitatively and qualitatively. The behavior of the CD8+ T cells responding to influenza infection was highly variable depending on the day after infection the imaging was performed. The most dramatic changes occurred after infectious virus was eliminated from the tissue, triggering a substantial shift in cell motility between days 8 and 9. Blocking peptide/MHC complexes with antibodies reversed cell arrest, increased velocities, and reduced confinement, similar to the changes observed from days 8 to 9. This suggested antigen-presentation persists after virus clearance with continued T cell engagement, and that T cell motility in the infected tissue is dynamically regulated by the infection and the presence of antigen-bearing cells in particular. In addition, these studies establish the trachea as a suitable site for live imaging of immune responses to virus infection.

## Introduction

Influenza virus productively infects the epithelial cells that line the upper and lower respiratory tract and is restricted to this site by a requirement for a locally-expressed trypsin-like enzyme that activates the viral hemagglutinin (HA) protein. The specific epithelial cell types and location along the respiratory tree are further influenced by the specificity of the viral HA for sialic acid moieties on the surface of the cells [1]. Most seasonal human influenza viruses, for example, recognize alpha-2, 6 linked sialic acids expressed on cells higher in the respiratory tract [1]. Avian H5 influenza viruses favor alpha-2, 3 linked sialic acids expressed in birds and on cells lower in the human respiratory tract [2, 3]. This partially explains the poor transmission of the H5 avian influenzas in humans [2]. Viral replication deep in the lung and alveolar spaces is associated with severe disease and is uncommon in most human infections with seasonal strains of the virus, which are restricted to the trachea, upper airways, and nasopharynx [4–7]. In general, transmission of influenza occurs through the generation of aerosol droplets in the nasopharynx followed by airborne transfer [8] or contact with contaminated surfaces [9, 10]. It follows that viral replication and immune control in the nasopharynx and proximal trachea are important for influenza epidemics and pandemics. Early characterizations of influenza pathology in humans and in animal models describe an acute tracheitis [5, 7, 11], which is how many clinicians characterize the disease caused by seasonal flu. Yet many contemporary animal models of influenza immunity have focused on events in the lung, blood, or lymphoid organs, with few recent studies of the infection and immune response in the trachea, upper airways, and nasopharynx.

During a primary influenza infection, cytotoxic CD8+ T cells (CTL) are the main effectors mediating elimination of infected cells [12–14]. The CTL directly engage infected targets through class I MHC-viral peptide complexes expressed on the surface of infected cells [13, 15, 16]. Therefore, the CTL must have mechanisms that allow them to enter the epithelium and mediate effector function. However, in spite of the importance of migration into the airways, the spatial locomotive, chemotactic, and adhesion mechanisms that regulate this process are only partially understood. Most studies that have identified molecular cues have inferred the function of these molecules by measuring relative accumulation of cells in the airways (sampled by lavage), or lung tissue (disrupting the tissue to harvest lymphocytes), or by performing conventional microscopic examination of tissue sections after immunostaining to localize T cells. In order to more fully understand cellular immunity to influenza infection, studies of immune cell behavior and regulation in the tissue at the site of infection are needed. For example, using live imaging, we showed that neutrophil-derived CXCL12 is an important molecular cue that guides CD8+ T cells to the infected airways [17].

Many studies of experimental influenza infection and immunity rely on the mouse model, with investigations of cellular immune responses focused on those cells that can be easily obtained by bronchoalveolar lavage (BAL) sampling of cells in the airways, and/or mechanical disruption of the lung after dissection. For this reason, much of the literature about immune responses at the site of infection discusses events in the lung. However, with the exception of highly pathogenic influenza viruses, or in cases of immune suppression, influenza infection in humans is clinically characterized as an infectious tracheitis, with involvement of the large conducting airways. Influenza viruses that replicate high in the respiratory tree, including the trachea and nasopharyngeal epithelium, transmit more efficiently [18], so immune control of infection in the trachea may be more important to limit transmission. Therefore, we developed a mouse model of influenza infection of the trachea using the broncho-tropic H3N2 influenza virus A/Hong Kong/X31 for the study of immune responses [19]. Infection of the trachea is not a new observation, and early (>40 yrs ago) descriptions of animal models of influenza mention of the prominence of this site [4, 7, 20, 21]. Furthermore, the trachea offers a relatively easy site to access for the purposes of live imaging, and as we show below, is more uniformly involved than the lung during infection with high specific output of virus.

An acute virus infection is dynamic in terms of the kinetics of viral replication, the cellular immune response, and virus clearance [22]. Unlike some models of live imaging of T cell responses that focus on a single time point for reproducibility, the present study interrogates the cells across the entire acute phase of the infection. Experiments were performed to distinguish the presence of infected cells versus persisting antigen in regulating T cell dynamics and motility.

The significance of this study is derived from the focus on the key effector cell responsible for controlling influenza infection, a disease that kills hundreds of thousands of people each year worldwide. Understanding the molecular basis of T cell motility and migration into the infected airways has the potential to lead to novel ways to improve control of influenza infection and pathogenesis. This new knowledge will translate into many other infections that occur in different mucosal sites and epithelial surfaces.

## Materials and Methods

### Mice

C57BL/6J (B6) mice were purchased from Jackson Labs (Bar Harbor, ME) and used from 8–12 weeks of age. A colony of the OT-I transgenic mouse strain that expresses a TCR specific for the OVA SIINFEKL (OVA257–264) peptide presented in the context of H-2Kb (14) was crossed with a transgenic mouse expressing GFP under a chicken beta-actin promoter [23]. Both lines of mice were maintained at the University of Rochester Vivarium. All animals were housed in the University of Rochester Vivarium facilities under specific pathogen-free conditions using microisolator technology.

### Ethics statement

All experimental protocols were performed in accordance with the standards established by the United States Animal Welfare Act, as set forth by the National Institutes of Health guidelines. The experimental protocols were reviewed and approved by the University Committee for Animal Resources, number 101431-UCAR-2006-029R, and conducted in Association for Assessment and Accreditation of Laboratory Animal Care International accredited facilities.

### Influenza viruses and infection

The influenza H3N2 A/Hong Kong/X31 (X31) virus, and A/X31-OVA-I influenza virus that expresses the ovalbumin (OVA) 257–264 (siinfekl) peptide in the hemagglutinin viral protein [24] were grown and titered in embryonated chicken eggs and harvested as allantoic fluid preparations (Allan et al., 1990). Mice were sedated with avertin (2,2,2-tribromoethanol) prior to intranasal (i.n.) challenge with 10^5^ EID_50_ of X31 or 3 x 10^3^ EID_50_ X31-OVA-I in 30 μl of PBS.

### Cell Isolation and flow cytometry

Following cardiac perfusion of the mice with PBS, the trachea was canulated to just above the tracheal carina (first bifurcation of the airways) and bronchoalveolar lavage cells were collected by lavage with 1 ml HBSS three times. These were then resuspended in complete (+10% fetal bovine serum) minimal essential medium (cMEM) (Gibco, Grand Island, NY). Single-cell suspensions were prepared from spleen and lymph nodes by disruption in cMEM by Dounce homogenization, washed, and resuspended in cMEM. Trachea and lung were collected and disrupted using a tissue chopper, then digested using Collagenase Type II (Worthington). Lymphocytes were isolated on a Percoll (Sigma, St. Louis, MO) gradient. Viable cell counts were obtained by trypan blue exclusion.

Cells collected from trachea were pooled in like groups (3–5 trachea per group) and stained with various combinations of mAbs to CD8a (53–6.7), CD4 (RM4-5), CD49a (Ha31/8), CD3 (145-2C11), Live/Dead Aqua (Invitrogen), and CD19 (1D3). The conjugated mAbs were purchased from BD Pharmingen, eBioscience or BioLegend and are referenced in their current catalogs. Tetrameric complexes of H-2Db/influenza polymerase (PA)224–233 (DbPA), and H-2Db/influenza nucleoprotein (NP)366–374 (DbNP), were prepared by the NIH Tetramer Facility (Atlanta, GA). Data was collected using an 18-color LSR-II, and analyzed using FlowJo software (Tree Star).

### Tissue staining and preparation

To obtain tissue for immunohistology, mice were infected intranasally with HK/X31. At time of harvest, mice were injected with Avertin and placed in a surgical plane. The renal artery was severed and the mouse was euthanized via exsanguination. The chest cavity was opened and the lungs were perfused with PBS. The trachea was exposed and a canula was inserted and fixed in place with a suture. Using a 1ml syringe, 0.8ml of warmed OCT was instilled to inflate the lungs, and a suture was placed at the base of the trachea to maintain the inflation. The lungs were excised and placed in OCT, then immediately frozen by floating on top of liquid Nitrogen. The trachea was also excised, inserted into OCT and frozen as for the lungs. Tissues were wrapped in aluminum foil and stored in a -80°C freezer.

Sections from 5–10 μm were cut transversally using a Shandon LSE Cryotome Cryostat. They were mounted on pre-cleaned superfrost plus slides from VWR. Slides were immediately placed in ice-cold acetone for 10 minutes for fixation, removed and allowed to dry for 10 minutes. They were then placed in a slide box, wrapped in aluminum foil, and stored at -80°C until ready for staining.

For immunofluorescent staining, sections were removed from -80°C and thawed on ice for 10 minutes. They were then placed at room temperature and allowed to dry for 10 minutes. Residual OCT was removed and rehydration of tissues was accomplished by incubating slides for 10 minutes with 1ml of PBS. The Fc receptor was blocked with PBS-Tween20 (0.05%) containing anti-CD16/32 (BD Pharmingen 1:200), normal rat serum and normal goat serum (Jackson, 1:100). Sections were incubated for 15 minutes at room temperature in a humidified chamber. From this point, all washing and staining was done using PBS-Tween20.

Sections were washed for 5 minutes and stained with the following antibodies: Alexa Fluor 647 labeled rat anti-mouse CD8a (BioLegend; 1:200), FITC labeled Monotope–Influenza A Virus (Virostat 1:100) and unlabeled goat anti-type IV collagen (Southern Biotechnology Associates; 1:400) for 60 min. Sections were then washed and incubated with a secondary donkey anti-goat Cy-3 for 30 min. The sections were then washed and mounted using a cover slip and DAPI Fluoromount-G (Southern Biotech). Fluorescence microscopy was performed at 20X using a Nikon Eclipse TE2000E fluorescence microscope equipped with an X-Cite Series 120 fluorescence Illuminator and a Cool-SNAP HQ charge-coupled device camera (Roper Scientific) with NIS-Elements software. All imaging filters were from Chroma Technology Corp. (Bellows Falls, VT).

For whole mount, intact tracheas were excised from infected mice. The tracheas were cut in half longitudinally and placed in PBS/BSA 1% (PBA). Fc receptors were blocked with CD16/32 (BD Pharmingen, 1:100). Primary antibodies were added in various combinations in excess (1:50): CD31, CD8, CD19, CD4 PE, Class II, ICAM, LYVE-1, and CD45 conjugated to FITC, PE, Alexa Fluor 647, Cy3, PerCP and APC. The conjugated mAbs were purchased from BD Pharmingen, eBioscience or BioLegend and are referenced in their catalogs. Tissue sections were incubated in a cold room at 4°C, on a rocker, for 4 hours. They were washed with 4 ml of PBA for 5 minutes and then mounted on a clean glass slide in 100 μl of PBA under a cover slip.

Imaging was performed with an Olympus BX40 with fluorescent attachment, a Retiga 1300 Monochrome cooled 12 bit camera from Q-Imaging systems, and Image Pro Plus Version 5.0 from Media Cybernetics.

### Multiphoton imaging

Imaging was performed using an Olympus FV1000AOM-Multiphoton imaging system in combination with a Spectra-Physics MaiTai-HP Deep See fs Ti:Sa laser system. Fluorescence was collected with a water immersion objective Olympus XLMUMPlanFI 25x NA1.05 for high-resolution imaging or a Zeiss C-Apochromat 10x NA0.45 water immersion objective for imaging large fields of view (~1200x1200um2). Fluorescence was separated from the excitation light using a dichroic mirror / blocking filter combination (Dichroic Olympus 650LP; Blocking Filters FF01-680/SP, Semrock, Rochester, NY). The fluorescence was divided into two channels using an appropriate dichroic mirror (505DCXRU, Chroma). The laser-power and the laser-pulse-width were continuously monitored using a Pulse-Scout-Autocorrelator (Spectra-Physics) and a wavelength-calibrated laser-power power meter (1918-C. Spectra-Physics). All imaging data was taken at 12bit-depth and analyzed and visualized using Volocity (Perkin Elmer) and Imaris (Bitplane) software, respectively.

### Cell transfer and infection

A line of GFP+/OT-1+ animals was maintained and used as donors for adoptive transfer of C57BL/6 naïve animals prior to infection. Whole splenocytes were isolated, and 1x10^6^ cells contained in 200ul of DPBS were transferred via tail vein. 24–48 hours post transfer, the animals were sedated with 120 mg/kg of Avertin. Once animals were sedated and exhibited no pedal reflex, 30 μl of X31-OVA-1 (2.7x10^3^ EID_50_/ml) was administered intra-nasally. Animals were monitored until fully recovered from anesthesia, and then daily for weight-loss.

### Sedation and Anti-convulsives

Prior to surgery, the animal was anesthetized with pentobarbital (65 mg/kg). During imaging the level of anesthesia was maintained with isofluorane, administered at 0.5–2% as necessary based on heart rate. Pancuronium bromide (0.4mg/kg) was administered prior to imaging, to prevent movement of the imaging area.

### Surgical set-up

The hair was removed with a shaver from one hind leg, thigh to groin, exposing the skin for the MouseOX Plus sensor (Starr Life Sciences). The hair was removed from the thoracic area with scissors and/or a shaver. The animal was placed in a supine position, on a warming blanket, once surgical plane of anesthesia was determined by lack of both pedal and palpebral reflex. The coat was opened from below the chin to the top of the ribcage. The salivary glands were separated to reveal the muscles covering the trachea. Using round forceps, the muscles were separated to expose the trachea. The forceps were then inserted beneath the trachea to lift and separate it from the muscle and mouse body. A small flexible plastic support was placed in the space created by the forceps to permanently hold the trachea above and separated from the muscle, surrounding tissue and coat.

The animal was moved to the previously warmed stage. A small incision was made between the cartilage rings below the larynx. The steel cannula was inserted into the opening in the trachea until it reached just below the sternum. The cannula provided physical immobilization of the trachea. The cannula was secured on the stage with a support that holds it in position so it is correctly aligned with the trachea. It was held in place with 2 screws that prevent it from moving in transport, or during the attachment of the respirator. The forepaws were secured to the stage with surgical tape to maintain position of the mouse body on the stage. A few drops of saline were placed on the exposed tracheal tissue to prevent drying.

### Maintaining blood O_2_ saturation and temperature

The cannula was quickly attached to the Harvard Inspira ASV ventilator, and both 100% O_2_ and 0.5% isofluorane flow was started, according to mouse weight. The MouseOX Plus thigh sensor was attached to the exposed thigh and monitoring was started immediately. Oxygenation levels were maintained at >95% and heart rate ranged between 250 and 600 beats per minute. The rectal body-temperature was continuously monitored and maintained using a small animal temperature controller that is connected to a rectal probe and a feedback-regulated rodent heating pad. After achieving stable physiology, and verification of lack of both pedal and palpebral reflex, the pancuronium bromide (0.4 mg/kg) was administered, based on body weight. The saline covering the exposed trachea was blotted away and replaced with 0.05% agarose to seal the exposed area. Once the agarose was solidified, a support ring was placed over the imaging area and covered with a piece of plastic wrap. Approximately 5 ml of water was pooled over the imaging area to submerse the objective.

### OVA peptide-MHC (Kb/siinfekl) blocking studies

Prior to the start of surgery, 100μg of anti-H2 Kb/ siinfekl (25-D1.16) antibody [25] was injected via tail vein. Animals were then prepared for imaging, which took place either one or two hours after administering the antibody. For the imaging, the animals were maintained either at 37°C or 35°C as described in the results.

## Results

### Kinetics of influenza virus replication in the trachea

**Fig 1:**
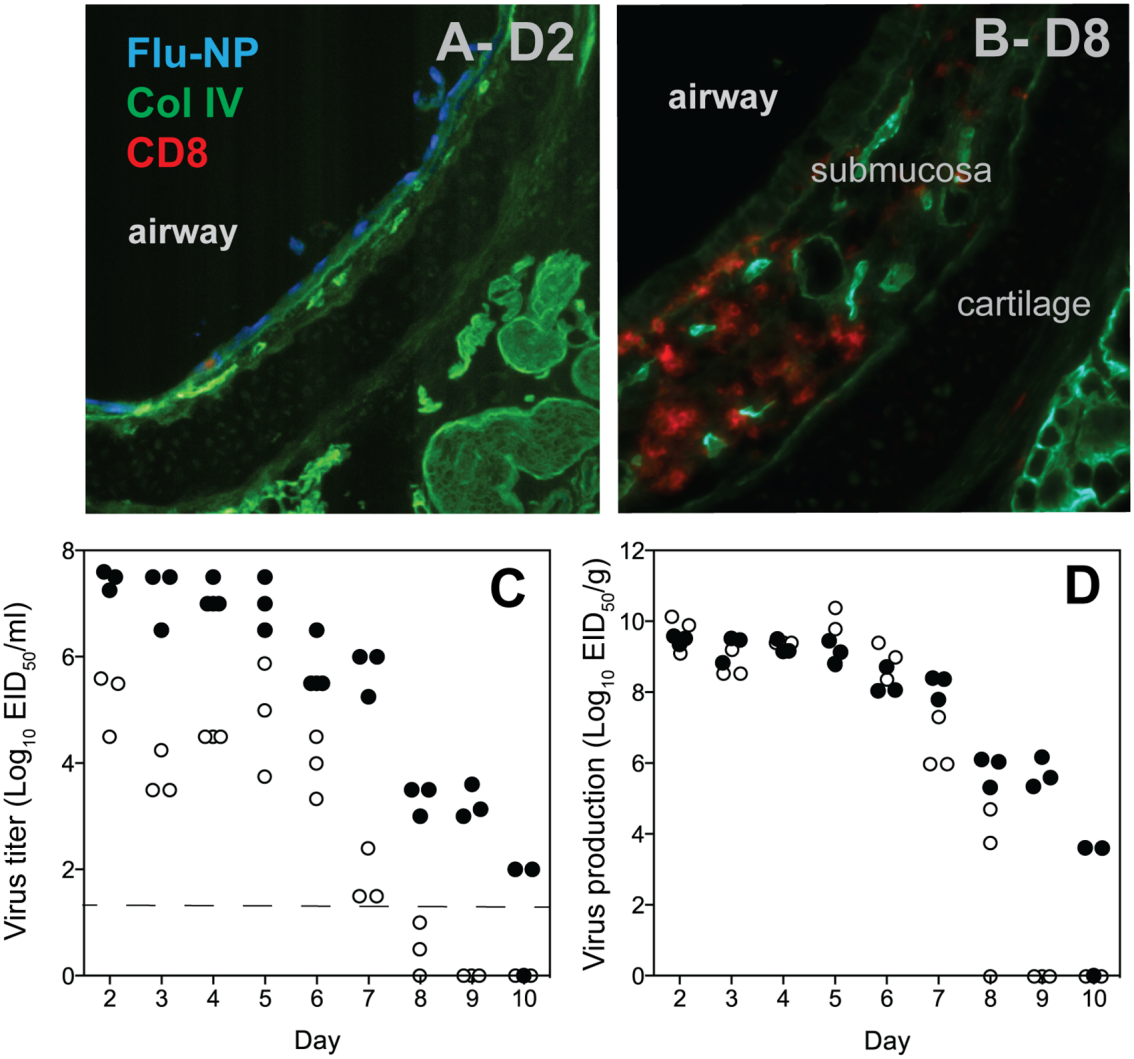
Influenza infection of the mouse trachea. C57BL/6 mice at 8 weeks of age were infected with 10^5^ EID_50_ of H3N2 influenza A/HK/X31 by an intranasal route. On days 2 and 8 the trachea was excised, snap frozen, and later sectioned and stained with antibodies to influenza nucleoprotein (NP, blue), collagen IV (Col IV, green), and CD8 (red); A = day 2, B = day 8. Separate cohorts of mice (n = 3–4) were similarly infected and virus titers in the trachea and lung were determined after homogenization of the tissues followed by dilution and inoculation of embryonated hen eggs (Methods). Titers of influenza virus in lung and trachea expressed as EID_50_ per ml of tissue homogenate (C) or per gram of tissue weighed prior to homogenization in 1 ml serum-free media (D). Closed circles = lung, open circles = trachea.

The well-established mouse model of non-lethal influenza infection has defined kinetics of viral replication in the lung, with clearance of the virus by 9 to 10 days after infection [26, 27], while T cell infiltration of the airways sampled by broncho-alveolar lavage (BAL) becomes easily detectable by day 6 [26, 28]. For the trachea, our initial goal was to determine if the kinetics of the virus and cellular immune responses were comparable to the BAL or lung tissue. To establish a baseline, mice were infected with a standard laboratory strain of H3N2 influenza A virus (HKx31) using an intranasal route of administration to sedated animals. Each mouse received 10^5^ EID_50_ of virus diluted in 30μl PBS [27, 29]. To determine the localization of infected cells and lymphocyte infiltrates, frozen sections were prepared for immunohistology and stained for CD4 and CD8 T cells, collagen IV, and influenza nucleoprotein (NP). On day 2 in mice infected with HKx31, the cells of the epithelium were uniformly positive for NP, with few T cells visible (Fig 1A). At day 8, the epithelium now demonstrated substantial T cell infiltrates in and below the epithelial surface (Fig 1B). This data suggests that HKx31 virus replicates efficiently in the tracheal epithelium.

To determine the kinetics of virus replication and production of infectious virus, the whole lung and a section of trachea (~3mm) were individually dissected from the animals, weighed, and snap frozen. When homogenized in an equal volume of media, the concentration of recovered virus in the trachea was lower than the lung at all time points, but followed a similar time course (Fig 1C). However, the amount of virus being produced per gram of tissue was equivalent to the lung (Fig 1D), despite the larger epithelial surface area. In the experiment shown, infectious virus was cleared from the trachea by day 9, and only one of three mice cleared virus from the lung by day 10, though the residual amount (~100 EID_50_) was low. Overall the kinetics of HKx31 are similar in trachea and lung tissue.

### Host cellular immune response in the trachea compared to lung tissue and airway

**Fig 2:**
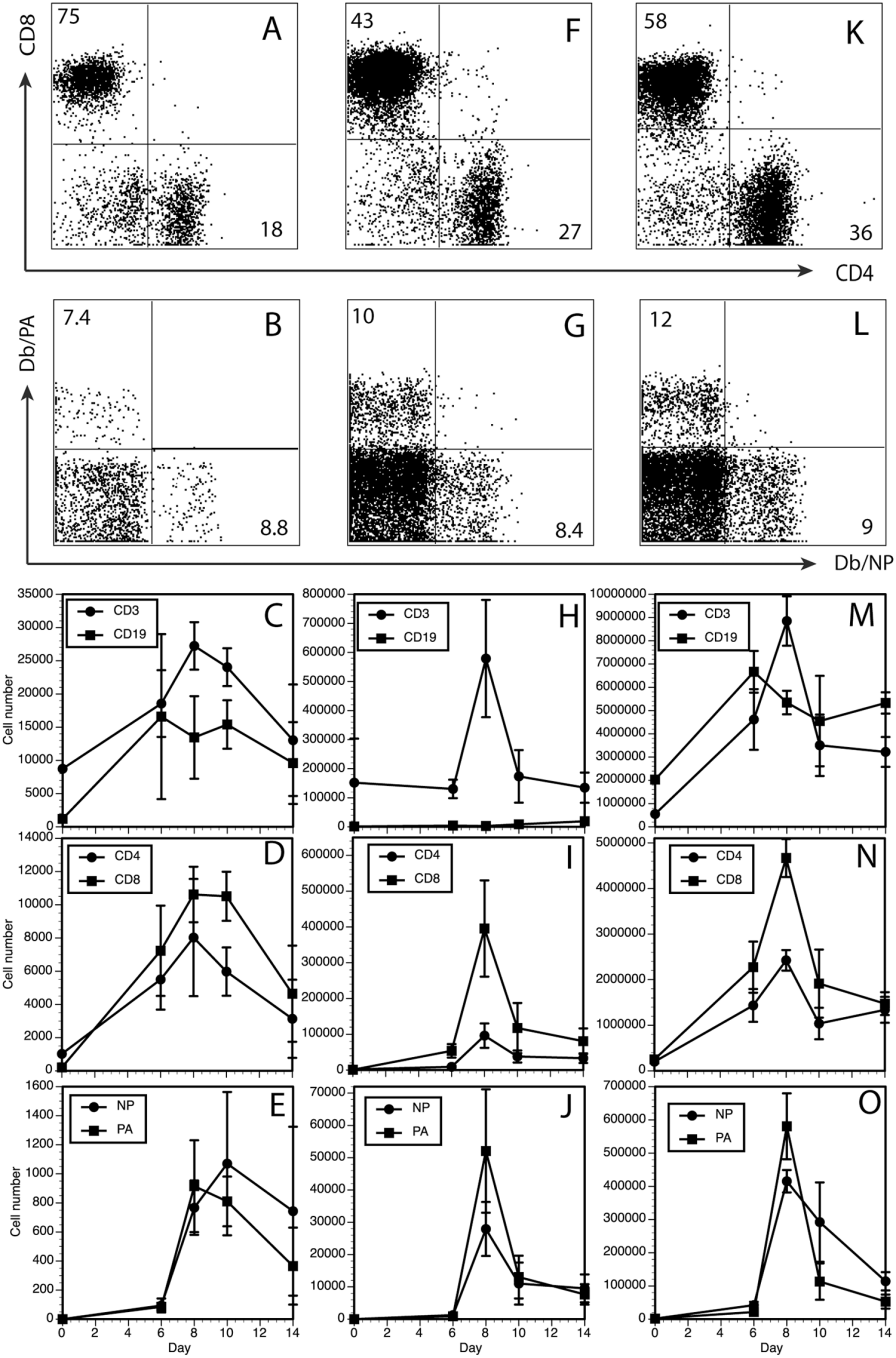
Cellular composition of influenza infected trachea, lung, and BAL. Mice were infected with influenza (as in Fig 1) and tissues harvested on different days after infection. Lung tissue was taken after broncho-alveolar lavage and perfusion of the blood vessels. Experiments consisted of pooled groups of 3 mice per time point, and 3–4 experiments per time point. Lung and trachea were enzyme digested and gently homogenized to make a single cell suspension and counted. Cells were stained with a panel of antibodies against T- and B-cell markers and MHC-Class I tetramers corresponding to immunodominant CD8+ T cell epitopes and analyzed by flow cytometry (Methods). Trachea = A-E, BAL = F-J, Lung = K-O. Each point represents the average of 3–4 experiments ± standard error of the mean.

Since the virus replication and clearance kinetics were similar in lung and trachea, we wanted to also compare T cell responses in the trachea to the airways sampled by BAL, and to lung tissue. Although the total cell numbers were small given the size of the tissue section (~10mg) and relative surface areas being sampled, CD4+ and CD8+, CD3+ T cells were easily detectable by flow cytometry on day 8 (Fig 2A, 2C and 2D), including Db/NP (8–11% of CD8+) and Db/PA (7–17% of CD8+) tetramer positive cells (Fig 2B and 2E). The trachea also contained higher proportions of CD19+ cells (5–10%) than the BAL (>1%), but less than the lung (~25%). Total CD3+ cells peaked 8 days after infection, and then declined. The peak of the response was less sharp than in the BAL or lung tissue (Fig 2), though it should be noted that the difference in cell numbers in the trachea from days 6, 8 or 10 are statistically insignificant, reflecting variability in cell recovery and a smaller denominator in terms of total cell counts. Collectively, these data show that the adaptive cellular immune response to influenza infection in the trachea is robust, with a rise and fall that corresponds with clearance of the virus as it does in the BAL and lung tissue.

### The trachea imaging site

**Fig 3:**
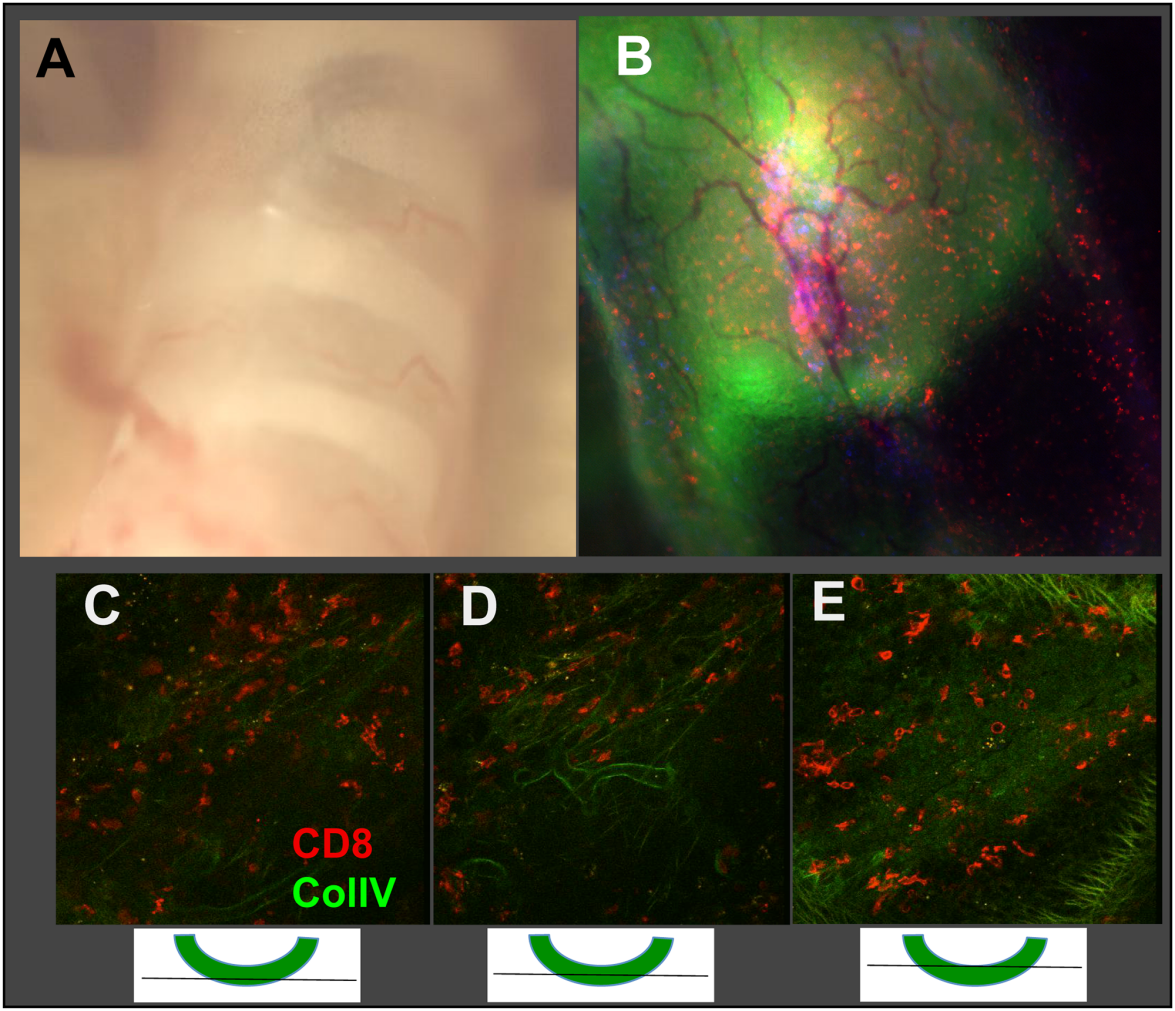
Intravital imaging site of the mouse trachea. B6 mice were infected as in Fig 1 or T cell receptor transgenic OT-I x GFP spleen cells (10^5^) were adoptively transferred into recipient B6 mice one day prior to infection with 3x10^3^ EID_50_ recombinant influenza A/HK/X31-OVA expressing the ovalbumin peptide siinfekl recognized by the OT-I T cells. **(A)** Exposed trachea just prior to imaging reveals its translucent appearance. **(B)** Whole mount imaging of excised trachea viewed from the inside. Tissue was stained with antibodies to CD8 (red) and Col IV (green). **(C-E)** Intact trachea from mice that received adoptive transfer of OT-I GFP+ cells, intravenous dextran (red) to label the blood vessels followed by imaging by multiphoton microscopy. Images C and D show 0 and 90 degree points of view through the trachea, with the SHG signal towards the outer wall of the trachea appearing in the distal layer of C and bottom of D.

Having established that the trachea is a suitable site of infection with HKx31 and X31-OVA, we proceeded to develop it as a site for imaging. To give some perspective on where in the tissue the imaging is performed, the trachea was surgically exposed in a live animal, showing it has a translucent appearance between the cartilage rings (Fig 3A). Imaging was performed on the tissue between the rings. For further perspective, explanted trachea was split longitudinally, opened and placed between a cover slip and glass slide for whole mount imaging. The tissue was stained for CD4 (blue) and CD8 (red) to image the T cells, and collagen IV (green) to visualize the lamina densa of the basement membrane, just underlying the epithelial surface (Fig 3B). Next, because of its ability to optically penetrate intact tissues to a greater depth, we used multiphoton imaging of the infected trachea to further reveal structural features and T cell localization. Dextran (red) was injected intravenously to highlight the blood vessels. Peering through the tissue, the blood vessels, T cells (green) and outer collagen fibers (white) can be visualized (Fig 3C–3E and S1 Movie). CD8+ cells are close to the blood vessels, in the parenchymal space below the epithelial surface. Unfortunately, the collagen bundles directly underlying the epithelium do not produce a detectable second harmonic signal. Using explanted trachea stained with antibodies to collagen IV, the T cells can be seen above and below the Col-IV layer corresponding to the lamina densa at the base of the epithelial surface, suggesting some of the T cells are in the epithelium itself (S1 Fig). This is consistent with conventional immunohistology using frozen sections [30]. The ability to optically penetrate the trachea from the outside in opens the possibility of imaging the infected trachea *in situ* in an intact live animal.

### Intravital multiphoton microscopy (IV-MPM) of lymphocyte migration in an intact, influenza infected trachea

**Fig 4:**
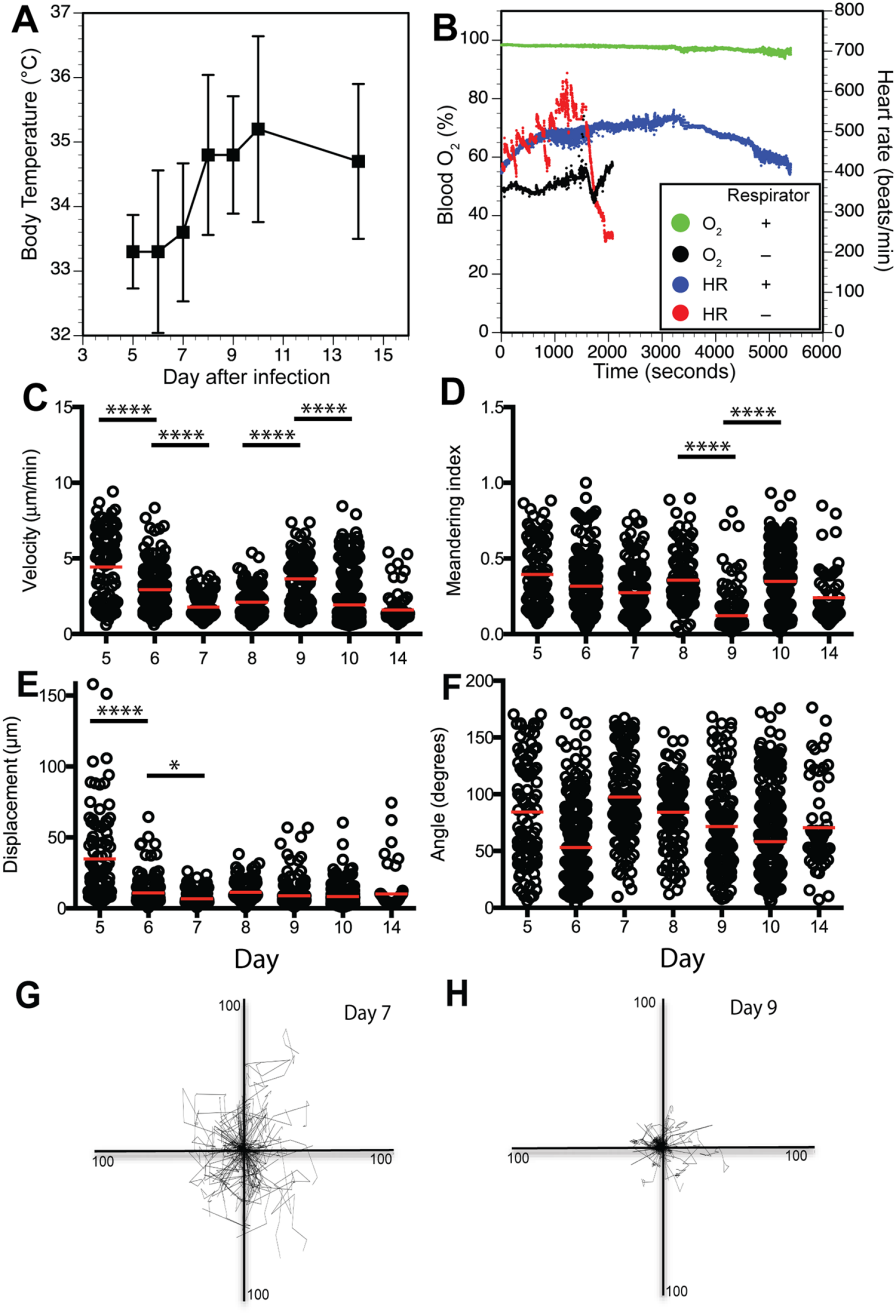
Physiological and motility parameters measured during intravital imaging of influenza infected mouse tracheas by multiphoton microscopy. Mice that received adoptively transferred GFP+ OT-I spleen cells and subsequently infected with X31-OVA-I virus as in Fig 3 were prepared and imaged on sequential days after infection by intravital multiphoton microscopy (Methods). **(A)** Rectal body temperatures of infected mice measured after sedation just prior to imaging. Each point is the average of 3–5 mice ± standard deviation. **(B)** Pulse-oximetry measurements of heart rate (HR) and percent blood oxygenation with and without respiratory support. Green and blue symbols represent blood O_2_ and HR, respectively, in a mouse on respiratory support. Black and red symbols represent O_2_ and HR, respectively from a mouse not receiving respiratory support. **(C-F)** Cell motility parameters calculated using Volocity software (Perkin-Elmer). Each symbol represents a single cell, and the plots contain 3–5 replicate experiments on individual mice. Horizontal gray bars denote the mean. C = velocity; D = Meandering index (also called confinement ratio); E = Displacement (distance form origin) for an entire cell track; F = average turning angle for each imaging step in a track. **(G-H)** Spider plots showing individual cell tracks plotted from a common origin using data from days 7 and 9 after infection.

Migration into the infected epithelium is critical for CTL mediated control of infected cells. Mechanisms that regulate effector CD8+ T cell motility in the infected tissue are largely undefined, and studies need to be done in the intact tissue to begin interrogating the mechanisms. Live imaging of the lung in live animals is limited by the need for the lung to continue functioning in breathing, with movement of the tissue and the challenge of maintaining intrathoracic pressure during respiration as significant technical hurdles [31]. Mice infected with influenza have lower body temperatures and low blood oxygen saturation [32]. Anesthetization can further lower body temperature [33, 34] and suppress breathing, affecting blood oxygen levels [34]. In our experiments, rectal temperatures of infected mice after sedation for imaging were variable, and lowest at days 5–6 (Fig 4A), a time when virus titers are still relatively high and the T cell infiltrates are just becoming measurable. As the animals recover and virus titers were reduced, pre-imaging body temperatures rise, though were still lower than reported for unsedated animals (Fig 4A) [32]. Mice being imaged also exhibited low blood oxygen saturation and erratic heart rates (Fig 4B). Because of concerns about the effects of physiological variability on cell motility, we elected to provide respiratory and temperature support to maintain normal physiology since cell movement is affected by both temperature and oxygenation [35]. Body temperature was controlled via a heat block on the stage, a warming blanket with feedback from the animal using a rectal probe, as well as a warmed objective lens. A respirator maintained breathing rate and oxygenation, with pulse oximetry. This setup allows longer-term (2h) imaging. Tracheostomy and cannulation of the trachea both stabilized the tissue from respiratory movement and facilitated mechanical ventilation.

### CD8+ T cell motility varies over the course of infection

First, we wanted to know how variable T cell motility was over the course of an acute infection. The number of studies measuring CD8+ T cell motility during respiratory infection is limited. CD8+ T cell velocity during a model of influenza infection has been reported to be similar on days 6 and 8, with significantly higher velocities on day 10 [36]. These experiments were performed using an excised lung slice and combined imaging of lung tissue and airway versus airway alone. To get a more comprehensive set of T cell motility parameters, and compare motility in primarily an airway to a more heterogeneous tissue, we adoptively transferred OT-I TCR transgenic CD8+ T cells crossed to a mouse that expresses GFP under an actin promoter, and then infected mice with X31-OVA-I at a sublethal dose. Note that this dose was lower than for wild-type X31 because mice infected with higher doses were too fragile for long-term imaging. Animals were imaged between days 5 and 14.

Events in the lymph node and spleen related to T cell activation and differentiation initially occur between one and 3 days after infection [22, 28, 29, 37, 38], well before virus-specific T cells become detectable in the respiratory tract. Few cells were visible prior to day 5, so imaging was done on days 5, 6, 7, 8, 9, 10, and 14 (S1–S7 Movies show an extended focus, while S8–S12 Movies are animated to visualize the cells and tissue from different angles in 3D). Visible cells were still variable on day 5 in some experiments, so we include only motility parameters from day 5 experiments that had substantial T cell infiltrates. Each imaging time point was repeated 3–5 times in replicate experiments using separate mice conducted over a period of more than a year.

CD8+ T cell motility was not uniform throughout the infection. Cell velocity began relatively high at days 5 and 6 and decreased steadily, reaching a nadir (p < 0.001) at day 7–8, presumably as effector CD8+ T cells accumulate in the respiratory epithelium where the infected cells are located (Fig 4C, S4b and S5b Movies). Remarkably, at day 9 there was a significant (p < 0.001) increase in velocity that then returned to slower speeds by day 10 and beyond (p < 0.001) (Fig 4C, S4a & S4b, S5a & S5b, S6a & S6b Movies). The meandering index, a measure of the straightness or confinement of cell tracks that is the ratio of the displacement of a cell to the total length of the path that the cell has travelled [39], also changed dramatically (p < 0.001) from day 8 to 9 and 9 to 10, being lowest at day 9, suggesting the cells were more restricted in their movement (Fig 4D and S12–S16 Movies). Displacement was highest at days 5 and 6 (the only time points significantly different than the others), suggesting rapid infiltration of the infected tissue (Fig 4E). Differences in displacement and cell tracks are depicted in spider plots for days 7 and 9 (Fig 4G and 4H).

**Fig 5:**
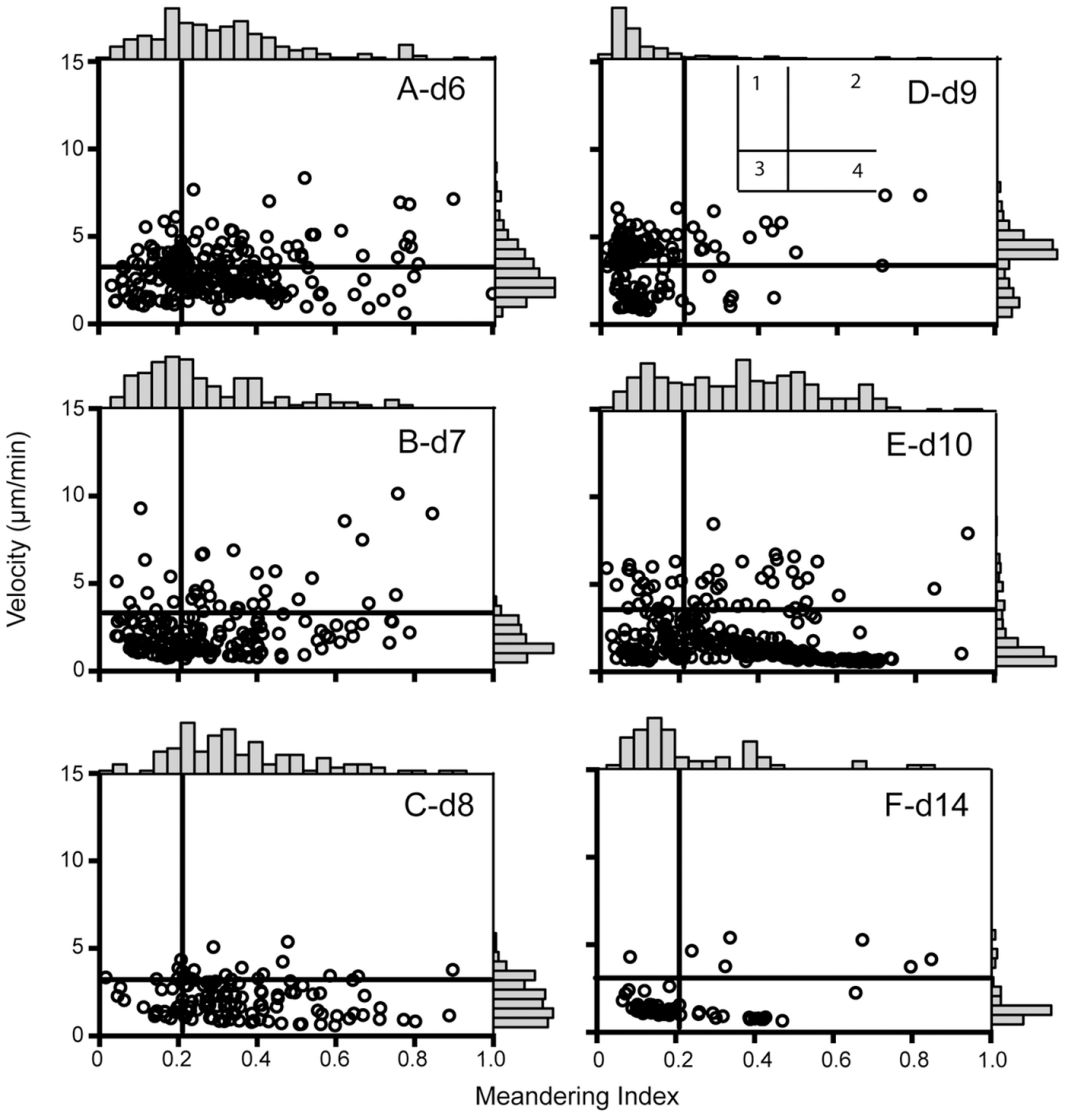
Meandering index versus velocity plots on different days after infection. The values for individual cells of meandering index and velocity are graphed to depict changing cell population behavior over the course of infection. Each symbol is data from a single cell. Histograms along the top and right axes indicate the cell densities. Crosshairs defining the quadrants are based on the densities in (D). A = day 6; B = day 7, C = day 8; D = day 9; E = day 10, and F = day 14.

**Fig 6:**
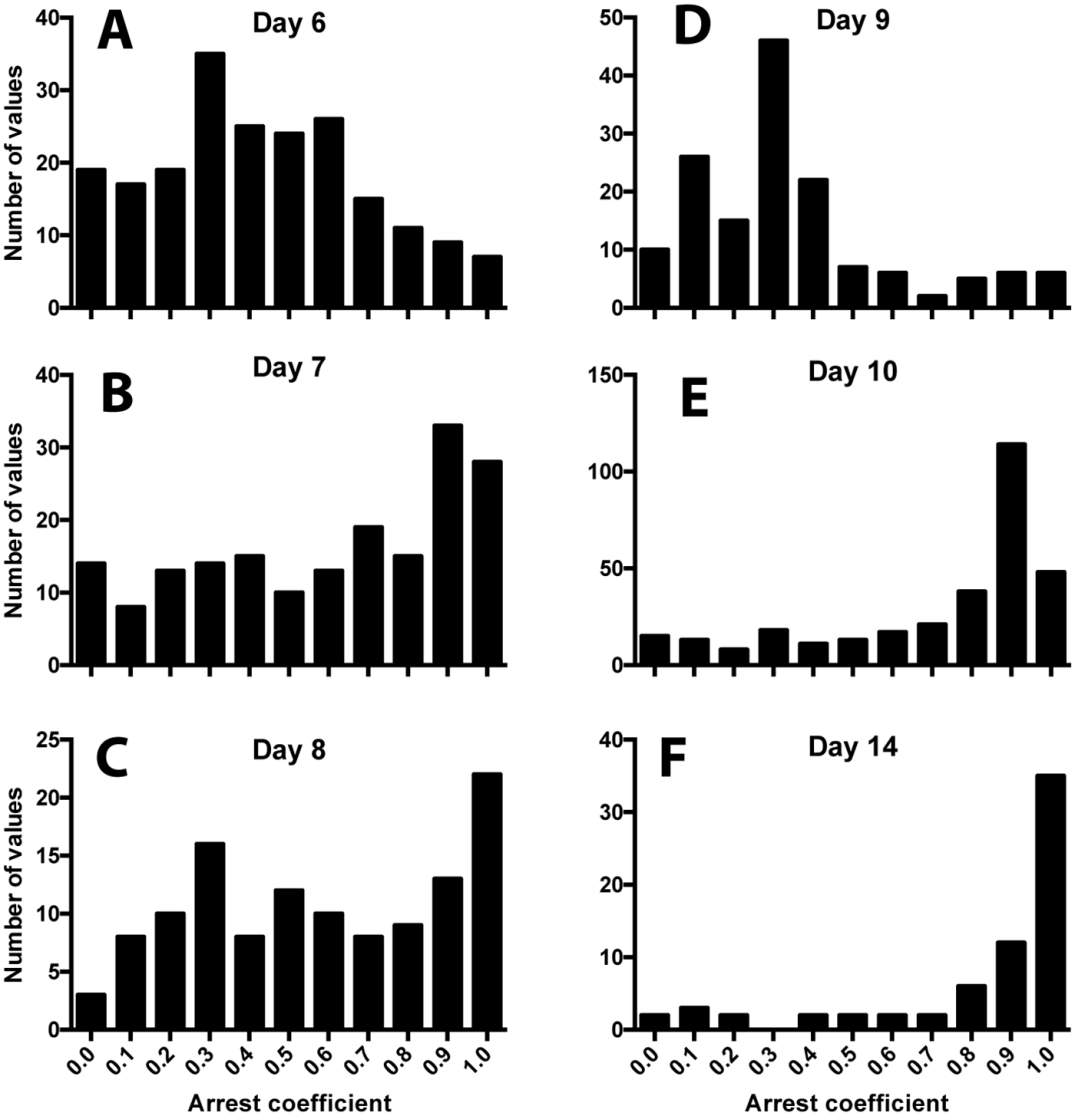
Frequency distributions of the arrest coefficients for each day after infection. For each day after infection measured, the arrest coefficients (defined as the fraction of time that a cell has a speed less than 2μm per minute) were calculated for individual cell tracks and the values of the population were binned into 0.1 increments. A value of 1 indicates full arrest. Y-axis indicates the number of coefficient values in each bin. A = day 6; B = day 7, C = day 8; D = day 9; E = day 10, and F = day 14.

To reinforce our interpretation of the motility data, we used an approach that provides a better visual representation of the changes in cell behavior. Since the cell motility parameters for each cell are linked, average cell velocities can be plotted against the meandering index to give an assessment of migratory properties over the course of the infection. Mrass et al. used this approach to describe four quadrants in the plot into which the cell populations fall (see inset Fig 5) [39, 40]. Quadrant 1 contains cells with high velocity and high apparent confinement (low meandering index); these are the cells exhibiting active directional migration, but that do not cover much distance from the origin. Quadrant 2 contains the cells with high velocity and low confinement, which are the cells showing sustained motility, with limited stopping. Quadrant 3 contains the cells of low velocity and high confinement, the so-called low motility or stopped cells. The fourth quadrant contains the cells with low velocity and low confinement, indicating cells that actively move only during certain periods, consistent with non-sustained motility. Histograms along the top and right side of each plot show the cell distribution. Similar to a flow cytometry plot, the locations of the crosshairs are based on the cell densities most easily seen in Fig 5D, but are otherwise arbitrary. The plots of velocity versus meandering index change rather dramatically over days 6–10 and day 14 (Fig 5). On day 6, cells are found distributed in all four quadrants, with many in quadrants 1 and 2, suggesting active directional and sustained motility (Fig 5A and S2 Movie). Fewer cells are in quadrant 3, exhibiting low motility. This is consistent with rapidly infiltrating cells that need to cover a lot of ground as they seek infected and antigen-bearing cells. On days 7–8 (Fig 5B and 5C), the majority of the cells move out of quadrants 1 and 2 (reduced velocity), and by day 8 most are in quadrant 4, exhibiting the non-sustained motility of cells that are not confined and actively moving during short periods (Fig 5C, S3 and S4 Movies). This behavior is consistent with cells that have reached their destination and are perhaps sequentially encountering antigen bearing and infected cells. On day 9 (Fig 5D), there is a major shift towards quadrant 1 and 3 as the velocities rise and the cells are moderately confined, consistent with low motility and displacement, showing that the cells are very active but not going very far. Closer examination of the d9 images shows many cells arrested and rounded in appearance compared to d8, while others exhibit rapid local migration (S13–S16 Movies). The high apparent velocities and increased confinement are consistent with rapid surveillance, perhaps sampling their immediate cellular environment for infection. On day 10 (Fig 5E) the cells shift again down to quadrants 3 and 4, which remains consistent on day 14, though with fewer cells (Fig 5F, S6 and S7 Movies). These are cells with low motility and high apparent confinement, possibly because they are entering a more sessile state. Calculation of the arrest coefficients supports these conclusions. On day 6, few T cells are arrested (Fig 6A). On days 7 & 8, the values are broadly distributed, with an increase in cells exhibiting arrest (Fig 6B and 6C), again perhaps as they accumulate at the epithelium. On day 9 few cells are arrested though their displacement values are low (Fig 6D), indicating that they are very actively moving, but not traveling far. On day 10, the majority of cells are close to arrest, while by day 14 the majority of cells are fully arrested (Fig 6E and 6F), which is not surprising given the absence of active infection at these time points.

### The effects of replicating virus and antigen availability on CD8+ T cell motility

**Fig 7:**
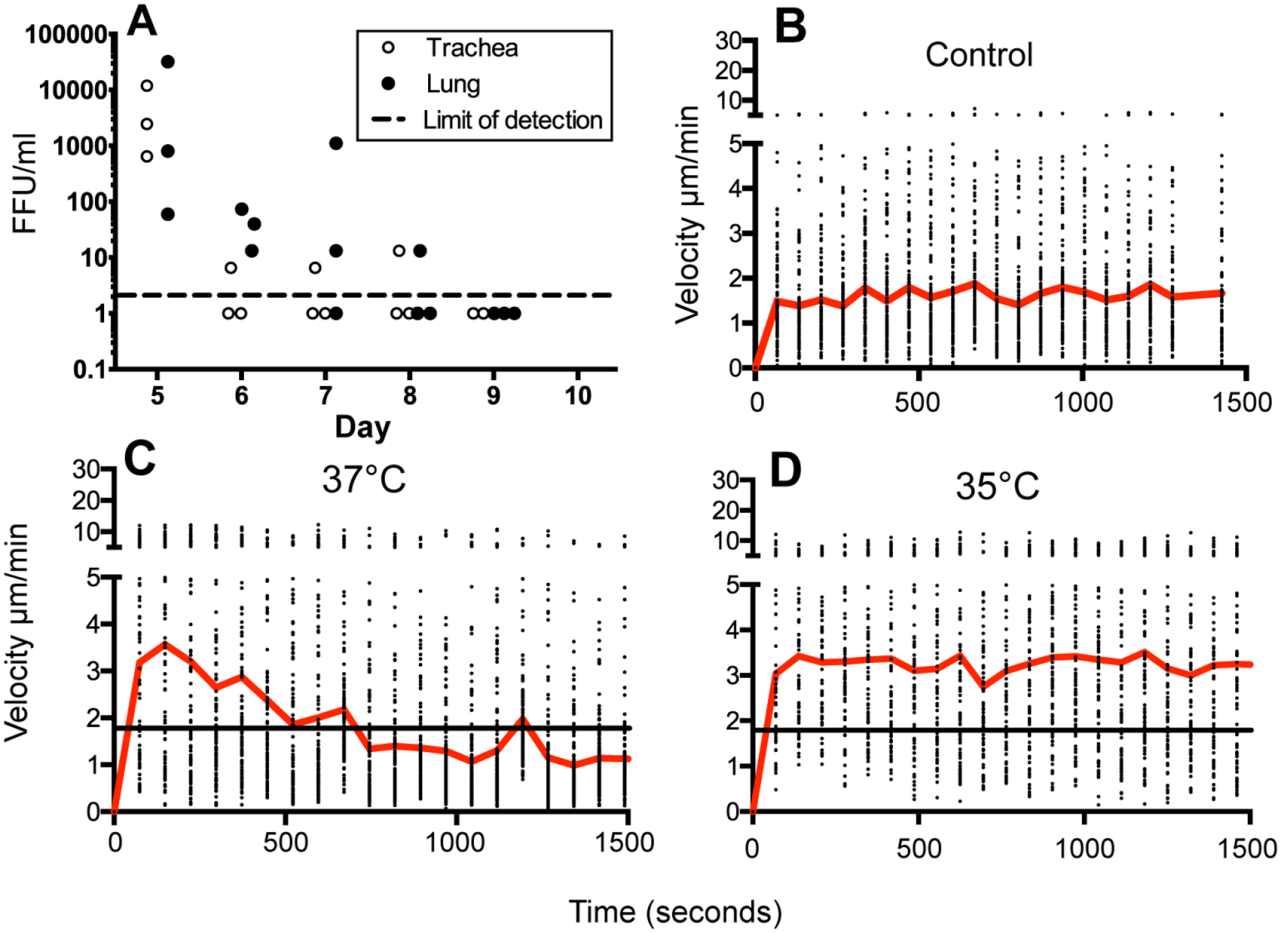
Virus clearance in mice infected with X31-OVA and the effect of OVA-peptide-MHC blocking antibodies on cell velocities. As described in Methods, OT-1-GFP CD8+ T cell receptor transgenic T cells were adoptively transferred into mice prior to infection with X31-OVA virus expressing the siinfekl OVA peptide. **(A)** Lungs and trachea were excised and titered by indirect immunofluorescence assay **(B-D)** The day 7 velocities of OT-I-GFP T cells are plotted over time during a 25-minute long imaging session. Control mice **(B)** were untreated. Experimental mice were treated with monoclonal antibody 25-D1.16 against Kb-siinfekl peptide-MHC complex, and imaged 2h later at 37°C **(C)** or at 35°C **(D)** with the warming objective turned off. The red lines indicate the average velocities at each increment over time, while the black horizontal line in **(C)** and **(D)** indicates the overall average of the day 7 control mice.

A substantial infiltrate of OT-I T cells in mice infected with influenza encoding the cognate siinfekl OVA H2Kb restricted peptide is present by day 5 post infection [24, 27]. To determine whether there were differences in virus clearance compared to non-OVA HKx31, we performed virus titers of lung and trachea at several time points after infection using an indirect immunofluorescence assay. Possibly due to the more rapid T cell response and lower dose of virus, virus titers were lower in the lung and below detection in the tracheas of two out of three mice at days 6, 7, and 8. However, one mouse was positive on all three of those days. The incomplete control may relate to a requirement for neutralizing antibodies, which are not typically detectable until day 7 [41]. We conclude that while virus was below the limit of detection in some mice, the data suggest that it may persist as far as day 8, which is similar to the wild-type kinetics. (Fig 7A)

Collectively, the data show that, early in the response, the cells travel at higher speed over more distance. As the cells accumulate along the epithelium, the displacement decreases and confinement increases, presumably due to physical restrictions to movement at the epithelial surface. The data also indicate an abrupt shift in motility that occurs between days 8, 9, and 10. The increase in velocity, decrease in angle and meandering index would be consistent with the cells becoming established in the epithelium, and exhibiting behavior associated with surveillance for residual antigen [42, 43]. To test whether this could be the case, mice were treated on day 7 with a monoclonal antibody that specifically blocks Kb/siinfekl peptide-MHC complex, while leaving other Kb class I complexes intact [44]. CD8+ T cell motility was measured 1 and 2 hours later. On day 7 in control mice, average CD8+ T cell velocities hover close to 2 μm/min (Fig 7B). In the antibody treated mice however, initial cell velocities at the start of the imaging session were between 3 and 4 μm/min, suggesting the antibody was having an effect (Fig 7C). However, over the 25-minute imaging session, the velocities returned to the lower values more closely resembling day 7–8 in untreated mice. We wondered whether the imaging conditions that include warming both the mouse body and the imaging site to 37°C with a warming objective was having an effect on the peptide MHC blockade. To test, we performed the imaging at 35°C, and turned off the objective lens warmer. Under the lower temperatures, cell velocities remained higher at between 3–4μm/min (Fig 7D). Both the slow decline at 37°C, and the stable cell behaviors at 35°C were reproduced in multiple mice on multiple imaging sessions. The data suggests that the low velocities and increased confinement on day 7, similar to day 8, is a result of cells arresting through interaction with antigen-bearing cells. The increased velocity on day 9 is most likely due to the elimination of the majority of the antigen-bearing cells (and virus). After day 10, the cells return to slower velocities, are less confined, with many cells arrested (velocities < 2μm/min). Given that the virus is absent at this and later time points, it is most likely a reflection of a less activated phenotype.

## Discussion

Utilization of the mouse trachea to study influenza infections is not a new idea, as infection of the trachea was described as long ago as the 1960’s and perhaps earlier [5, 7, 20]. Natural infection of humans with circulating seasonal influenza virus strains is similarly clinically presented as tracheitis [45], except in extreme cases of highly pathogenic strains or immunosuppression leading to more widespread infection of the lung [45]. The study described here, using a mouse-adapted strain of influenza (H3N2 influenza A/Hong Kong/X31) [46], shows that tracheal epithelium is uniformly infected as demonstrated by influenza nucleoprotein positive cell staining within 2 days of inoculation. Specific production of virus by the tracheal tissue is high and on par with the lung as a whole. The data show that the trachea is a very relevant site in which to study the antiviral immune response.

The majority of studies in the field of mouse models of influenza infection over the last 20 or so years have largely focused on events in the lung as a whole. Typical study designs, including many from our lab, involved collecting bronchoalveolar lavage to sample the airways, and explanting the entire lung followed by tissue disruption to make single cell suspensions of infiltrating lymphocytes for *in vitro* assays. Because the preponderance of recent quantitative cellular immunity literature is derived from the lung, not the trachea, we felt that it was important to provide a complete characterization of the cellular response to influenza infection of the trachea. The data was useful in designing the imaging studies that follow. Not surprisingly, the initial immune response to primary infection stimulates a cellular response limited by the time it takes to prime T cells in the draining lymph nodes and spleen, cellular proliferation, and eventual circulation via the blood to sites of infection. As observed in the lung, this takes up to 4–5 days before substantial numbers of virus-specific CD8+ T cells can be detected either by tissue disruption or direct imaging methods.

The imaging of an immune response in a respiratory tissue is not without its challenges. The principal technical hurdle is overcoming the physiological requirement of the lung to expand and contract for breathing and gas exchange. While there are algorithms that can correct for small tissue movement, the degree to which the lung moves in a single breath cycle exceeds the capability to correct for motion [31]. Strategies that have been used by others to overcome this issue include using lung explants of mostly intact tissue [36], and thoracic “windows” in which the lung is suctioned against a clear viewing portal placed by cutting through the chest wall [32]. To our knowledge, only the former approach has been used in a model of influenza infection. We succeeded in targeting the trachea with a combination of minor surgery, optimization of paralytic and sedative agents, and partial physical immobilization by cannulation. Combined with transgenic reporter cells (OT-I-GFP) and recombinant virus that encodes the cognate OVA peptide [24, 27], we have demonstrated that the trachea is an optically penetrable tissue suitable for cell migration studies.

A finding that was both unexpected at the time and, in retrospect, unsurprising, was that the influenza-infected mice prepared for imaging were physiologically compromised and fragile. This was caused by the combination of the consequences of the infection and the use of anesthetics on the mice, both of which can reduce respiration and blood oxygen saturation [33]. In a study reported in 2009, Verhoeven and Farber [32] demonstrated that pulse oximetry to measure blood oxygen was a more sensitive measure of lung pathology than the more widely used weight loss measure. Blood oxygen levels declined with increased virus dose (and titers) as well as with the presence or absence of an adaptive immune response, which when absent caused reduced weight loss, but much lower oxygen saturation and increased lung pathology [32]. These authors went on to investigate the effects of warming on blood oxygen, and concluded there was no effect. Although we did not directly investigate this ourselves, temperature can affect cell migration independent of its effects on blood oxygen [47]. T cells move optimally at a normal physiological temperature [47]. Given the reduced body temperatures induced by both the infection and sedation, we felt compelled to maintain the body temperature at close to normal healthy levels. One could argue that we should have maintained a temperature closer to that of a “normal” flu infected animal, which is reported to be an average of 3.8°C below normal [32]. However, we reasoned that it was best to maintain a common baseline temperature in all the experiments to reduce the chance of introducing additional mouse-to-mouse and day-to-day variability to the motility data. Therefore mechanical respiration and feedback controlled temperature maintenance became part of our standard protocol for imaging.

Studies of CD8 T cell motility in the airways or lung tissue are limited compared to those on neutrophils [31], dendritic cell (DC) behavior and migration [36, 48], or CD4+ T cell migration in models of allergic airway disease [48]. In our studies, we found that CD8+ T cells migrated at the highest apparent velocities on days 6 and 9, the two time points that frame initial T cell infiltration of the tissue and the time when virus is cleared. However, analysis of confinement at those two time points revealed markedly different values that demonstrate the T cells are far more confined on day 9 and do not migrate far from their origins. During the acute phase when virus was still present, the CD8+ T cells moved at the slowest average velocities and moderate confinement at day 8, consistent with sequential engagement of antigen bearing cells and/or localization in the epithelial layer, which would be expected to limit lateral migration. Indeed turning angles clustered around the 90° point at day 8 which would be consistent with movement in the relatively two-dimensional space of the epithelium. After the virus was cleared, cell velocities returned to lower values, suggesting a less activated state. Interestingly, day 10 is a time point that we have observed that the CD8+ T cells in the airways become dependent on the integrin alpha-1 for retention [30, 49, 50]. A similar phenomenon of slowed migration was noted recently in explanted lung tissue from infected mice, where the authors also suggested this is a time when the cells begin rapid migration on collagen [36], consistent with our hypothesis that this is when the T cells switch to an integrin-dependent mode. Notably, they also observed higher velocities on day 10, which is the day virus (and possibly antigen) is cleared in the lung, and potentially similar to the phenomenon we saw on day 9 in the trachea. It is also possible that the T cell populations in the tissue from days 8, 9, 10, and 14 are not the same, as the airway is not a closed system. Our data on the role of alpha-1 integrin suggest that, at least after virus clearance, there is a selective process for the cells that can be retained [49, 50], possibly leading to the establishment of tissue-resident memory, which if true may account for the differences in cell motility behaviors.

Compared to the aforementioned study using explanted lung sections [36], our data from the trachea of influenza-infected mice differs in that we see lower (vs. higher) cell velocities on day 8 than day 6. The day 6 CD8+ T cells were equally moderately confined and average velocities were similar (not lower) on day 14 compared to day 10. It is possible that there is a difference in overall cell and viral clearance kinetics that can account for these variations. For example, if you use day 9 in our studies to compare to days 8 and 10, you get a set of observations more similar to the differences noted in the lung comparing day 8 to days 6 and 10. Interestingly, both studies note the brief switch to a rapid scanning mode on the day after virus clearance has been demonstrated.

Other mechanisms could also account for the differences in CD8+ T cell motility behavior on different days of the acute response. We recently demonstrated that neutrophil-derived chemokine CXCL12 deposited in membrane vesicles onto the extracellular matrix stimulates CD8+ T cell chemotaxis sensed through CCR4 on the CD8+ T cells in the flu-infected trachea [17]. Inhibition of this mechanism through various interventions reduced the rate of CD8+ T cell infiltration into the tissue (measured at day 8 or earlier), by affecting cell velocities and localization within the tissue relatively distal to the airway surface [17]. It is likely that this is an effect manifested at the early time points when the T cells are first infiltrating the tissue, and diminishes towards the end of the acute response as the neutrophil vesicles become less frequent. The higher apparent velocities in neutrophil-depleted mice is consistent with the interpretation of the data in the current paper that progressive localization into the epithelial layer and engagement of antigen-bearing cells from days 6 to 8 slows the T cells.

There is a major contraction of the CD8+ T cell response between days 8 and 9, with massive apoptosis of the T cells [49]. However, we do not think the remaining cells measured on day 9 are apoptotic given their apparent high velocity readings that indicate the cells must be viable, as well as our earlier data that shows no measurable increase in the proportion of apoptotic cells [49]. The blip in cell velocities and increased confinement we observed between days 8 and 9 is remarkable. We wondered whether it was the infection itself or the presence of antigen-bearing cells that changed. Experiments were performed to distinguish antigen from infection. While some mice in the OT-I/X31-OVA-I system may have cleared virus as early as day 6, it was treatment with OVA-peptide MHC blocking antibodies that altered cell motility, increasing their velocities. The resemblance of these increased velocities to that observed on day 9 suggests that the event driving the transient increase on day 9 reflects clearance of most antigen-bearing cells. It is entirely conceivable that the CTL do not distinguish infected versus antigen-bearing cells and kill them, as antigen-bearing dendritic cells are eliminated by cellular cytotoxicity [12, 51]. Sensitive techniques to detect antigen-bearing cells have not been developed, making it difficult to measure the presence or absence of these cells directly. We also did not identify the phenotype of the antigen bearing cells.

Obvious follow-up studies that need to be performed include the study of dendritic cells and monocyte/macrophage, and role of these cells in regulating cell motility and localization. It will be critical to investigate the role of extracellular matrix interactions through integrins and other matrix receptors that are known to affect the CD8+ T cell responses to flu [40]. The fact that the peptide-MHC antibodies seemed to get into the tissue when administered intravenously appears to rule out the possibility that the integrin antibodies would not get to the site, making blocking studies, such as those done in the inflamed dermis [52], feasible. Similarly, studies looking at dependence on chemokine receptor signals at all stages of the response need to be performed. It will also be important to look at the memory phase of the immune response to influenza in the trachea, as well as secondary infection, which is the scenario closest to most human infections.

In closing, we have presented a comprehensive analysis of using the mouse trachea as a site to study the migration behavior and mechanisms regulating cell motility. We have demonstrated its relevance to the overall cellular immune response to primary infection, and as an important site of virus replication. The behavior of the CD8+ T cells we measured differed in several aspects from that reported in other sites of influenza infection. Interference with OVA peptide/MHC complexes suggests the T cells in the influenza infected tissue are affected by antigen-bearing cells, providing an explanation for slowed velocities and high confinement observed just prior to virus clearance. The ability of the cell to increase velocities on day 9, or under the influence of peptide-MHC blocking, tells us that the cells are not necessarily physically confined by their environment. We believe this work contributes to a better understanding of influenza immunity and will lead to novel interventions that could improve the control of virus infection, establishment of protective memory, and reduction of the tissue damage caused by the infection and immune pathology.

## Supporting Information

S1 Fig Multiphoton microscopy of day 8 X31-OVA infected trachea with OT-I CD8+ T cells. Explanted trachea was stained for Col4 (green) and CD8 (red), then imaged by multiphoton microscopy. CD8+ T cells are visible in the space “above” the blood vessels (green) and on both sides of the Col4+ basement membrane (green) that is the basal surface of the epithelium. CD8+ T cells above the Col4 layer are likely in the epithelium itself.
(TIF)

S1 Movie Animated 3D image reconstruction of trachea showing relative localization of CD8 T cells, blood vessels, and second harmonic generation from the outer collagen sheath of the day 7 flu-infected trachea. Adoptively transferred OT-I-GFP CD8+ T cells (green) are imaged by multiphoton intravital microscopy 7 days after infection with influenza X31-OVA-I as described in materials and methods. The blood vessels are imaged after injecting anti-CD31-PE intravenously (red). Outer collagen sheath provides an optical signal through second harmonic generation (white).
(MP4)

S2 Movie Extended focus videos of day 6 CD8 T cell motility in the flu infected trachea. Adoptively transferred OT-I-GFP CD8+ T cells (green) are imaged by multiphoton intravital microscopy after infection with influenza X31-OVA-I as described in materials and methods. The movies show examples of the extended focus videos, taken from a point of view inside the trachea peering through, used to generate the motility parameters for days 6–10 after infection.
(MP4)

S3 Movie Extended focus videos of day 7 CD8 T cell motility in the flu infected trachea. Adoptively transferred OT-I-GFP CD8+ T cells (green) are imaged by multiphoton intravital microscopy after infection with influenza X31-OVA-I as described in materials and methods. The movies show examples of the extended focus videos, taken from a point of view inside the trachea peering through, used to generate the motility parameters for days 6–10 after infection.
(MP4)

S4 Movie Extended focus videos of day 8 CD8 T cell motility in the flu infected trachea. Adoptively transferred OT-I-GFP CD8+ T cells (green) are imaged by multiphoton intravital microscopy after infection with influenza X31-OVA-I as described in materials and methods. The movies show examples of the extended focus videos, taken from a point of view inside the trachea peering through, used to generate the motility parameters for days 6–10 after infection.
(MP4)

S5 Movie Extended focus videos of day 9 CD8 T cell motility in the flu infected trachea. Adoptively transferred OT-I-GFP CD8+ T cells (green) are imaged by multiphoton intravital microscopy after infection with influenza X31-OVA-I as described in materials and methods. The movies show examples of the extended focus videos, taken from a point of view the trachea peering through, used to generate the motility parameters for days 6–10 after infection.
(MP4)

S6 Movie Extended focus videos of day 10 CD8 T cell motility in the flu infected trachea. Adoptively transferred OT-I-GFP CD8+ T cells (green) are imaged by multiphoton intravital microscopy after infection with influenza X31-OVA-I as described in materials and methods. The movies show examples of the extended focus videos, taken from a point of view inside the trachea peering through, used to generate the motility parameters for days 6–10 after infection.
(MP4)

S7 Movie Extended focus videos of day 14 CD8 T cell motility in the flu infected trachea. Adoptively transferred OT-I-GFP CD8+ T cells (green) are imaged by multiphoton intravital microscopy after infection with influenza X31-OVA-I as described in materials and methods. The movies show examples of the extended focus videos, taken from a point of view outside the trachea peering through, used to generate the motility parameters for days 6–10 after infection.
(MP4)

S8 Movie 3D animation of day 6 CD8 T cell motility in the flu infected trachea. Adoptively transferred OT-I-GFP CD8+ T cells (green) are imaged by multiphoton intravital microscopy after infection with influenza X31-OVA-I as described in materials and methods. Rotating 3D images were reconstructed from the extended focus videos to show the lateral point of view of the tissue to illustrate depth in the localization of T cells (green) relative to the outer collagen sheath (white).
(MP4)

S9 Movie 3D animation of day 7 CD8 T cell motility in the flu infected trachea. Adoptively transferred OT-I-GFP CD8+ T cells (green) are imaged by multiphoton intravital microscopy after infection with influenza X31-OVA-I as described in materials and methods. Rotating 3D images were reconstructed from the extended focus videos to show the lateral point of view of the tissue to illustrate depth in the localization of T cells (green) relative to the outer collagen sheath (white).
(MP4)

S10 Movie 3D animation of day 8 CD8 T cell motility in the flu infected trachea. Adoptively transferred OT-I-GFP CD8+ T cells (green) are imaged by multiphoton intravital microscopy after infection with influenza X31-OVA-I as described in materials and methods. Rotating 3D images were reconstructed from the extended focus videos to show the lateral point of view of the tissue to illustrate depth in the localization of T cells (green) relative to the outer collagen sheath (white).
(MP4)

S11 Movie 3D animation of day 9 CD8 T cell motility in the flu infected trachea. Adoptively transferred OT-I-GFP CD8+ T cells (green) are imaged by multiphoton intravital microscopy after infection with influenza X31-OVA-I as described in materials and methods. Rotating 3D images were reconstructed from the extended focus videos to show the lateral point of view of the tissue to illustrate depth in the localization of T cells (green) relative to the outer collagen sheath (white).
(MP4)

S12 Movie 3D animation of day 10 CD8 T cell motility in the flu infected trachea. Adoptively transferred OT-I-GFP CD8+ T cells (green) are imaged by multiphoton intravital microscopy after infection with influenza X31-OVA-I as described in materials and methods. Rotating 3D images were reconstructed from the extended focus videos to show the lateral point of view of the tissue to illustrate depth in the localization of T cells (green) relative to the outer collagen sheath (white).
(MP4)

S13 Movie Video collected at day 8 at higher resolution (512p) for a closer, more detailed view of CD8 T cell behavior just prior to viral clearance. As above, adoptively transferred OT-I-GFP CD8+ T cells (green) are imaged by multiphoton intravital microscopy after infection. The animation shows the majority of relatively large cells actively migrating, with a few stationary cells having a more rounded appearance. Second harmonic signals removed.
(MP4)

S14 Movie Video collected at day 8 at higher resolution (512p) for a closer, more detailed view of CD8 T cell behavior just prior to viral clearance. As above, adoptively transferred OT-I-GFP CD8+ T cells (green) are imaged by multiphoton intravital microscopy after infection. The animation shows the majority of relatively large cells actively migrating, with a few stationary cells having a more rounded appearance. Second harmonic signals removed.
(MP4)

S15 Movie Video collected at day 9 at higher resolution (512p) for a closer, more detailed view of CD8 T cell behavior just after viral clearance. As above, adoptively transferred OT-I-GFP CD8+ T cells (green) are imaged by multiphoton intravital microscopy after infection. The animation shows cell that are mostly arrested in the tissue but actively reaching out presumably to contact cells in their immediate vicinity. Behavior is consistent with active surveillance of local cells. Second harmonic signals removed.
(MP4)

S16 Movie Video collected at day 9 at higher resolution (512p) for a closer, more detailed view of CD8 T cell behavior just after viral clearance. As above, adoptively transferred OT-I-GFP CD8+ T cells (green) are imaged by multiphoton intravital microscopy after infection. This animation shows an example of more actively moving T cells, with limited displacement in the tissue. Behavior is consistent with active surveillance of local cells. Second harmonic signals removed.
(MP4)
