# Supplementary figures and images for: Live Imaging of Influenza Infection of the Trachea Reveals Dynamic Regulation of CD8+ T Cell Motility by Antigen
Source: PLoS Pathog. 2016 Sep 19;12(9):e1005881. doi: 10.1371/journal.ppat.1005881 (PMC5028057; doi:10.1371/journal.ppat.1005881)

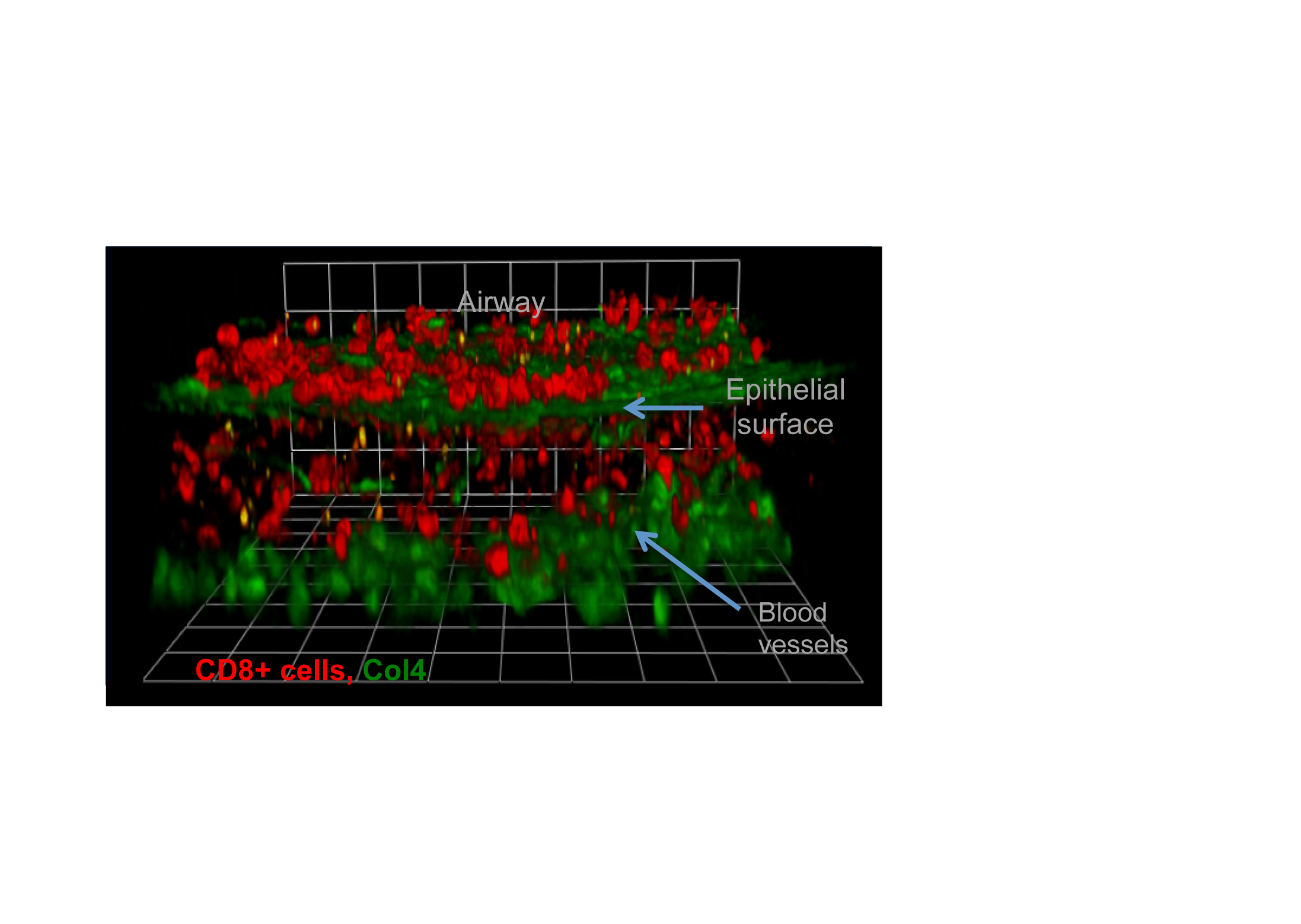

Supplement: S1 Fig — Explanted trachea was stained for Col4 (green) and CD8 (red), then imaged by multiphoton microscopy. CD8+ T cells are visible in the space “above” the blood vessels (green) and on both sides of the Col4+ basement membrane (green) that is the basal surface of the epithelium. CD8+ T cells above the Col4 layer are likely in the epithelium itself. (TIF) [file ppat.1005881.s001.tif]
